# Quantitative radiomics studies for tissue characterization: a review of technology and methodological procedures

**DOI:** 10.1259/bjr.20160665

**Published:** 2017-02

**Authors:** Ruben T H M Larue, Gilles Defraene, Dirk De Ruysscher, Philippe Lambin, Wouter van Elmpt

**Affiliations:** ^1^ Department of Radiation Oncology (MAASTRO), GROW—School for Oncology and Developmental Biology, Maastricht University Medical Centre, Maastricht, Netherlands; ^2^ Department of Oncology, Experimental Radiation Oncology, University of Leuven, Leuven, Belgium

## Abstract

Quantitative analysis of tumour characteristics based on medical imaging is an emerging field of research. In recent years, quantitative imaging features derived from CT, positron emission tomography and MR scans were shown to be of added value in the prediction of outcome parameters in oncology, in what is called the radiomics field. However, results might be difficult to compare owing to a lack of standardized methodologies to conduct quantitative image analyses. In this review, we aim to present an overview of the current challenges, technical routines and protocols that are involved in quantitative imaging studies. The first issue that should be overcome is the dependency of several features on the scan acquisition and image reconstruction parameters. Adopting consistent methods in the subsequent target segmentation step is evenly crucial. To further establish robust quantitative image analyses, standardization or at least calibration of imaging features based on different feature extraction settings is required, especially for texture- and filter-based features. Several open-source and commercial software packages to perform feature extraction are currently available, all with slightly different functionalities, which makes benchmarking quite challenging. The number of imaging features calculated is typically larger than the number of patients studied, which emphasizes the importance of proper feature selection and prediction model-building routines to prevent overfitting. Even though many of these challenges still need to be addressed before quantitative imaging can be brought into daily clinical practice, radiomics is expected to be a critical component for the integration of image-derived information to personalize treatment in the future.

## INTRODUCTION

The use of quantitative imaging has been an attractive field of research to overcome the subjectivity of visual interpretation. However, in all imaging divisions from radiology to nuclear medicine, the amount of quantification is still limited and the majority of clinical decision-making is based on visual assessment. A common quantification is performed by the response evaluation criteria in solid tumours^1^ that is based on the measurement of tumour size and frequently used for response assessment in oncology, whereas PET Response Criteria in Solid Tumours^2^ is making its introduction in the nuclear medicine arena to allow simple quantification of maximum uptake of a tracer [*e.g.* maximum standardized uptake value (SUV) or SUV peak].

Besides these simple quantification methods, diagnosis is also complemented visually by the appearance of lesions having different properties or patterns that are used to differentiate between benign and malignant lesions. These appearances (*i.e.* imaging features) are typically described visually and the radiologist interprets and selects suspected lesions for future clinical investigations (*e.g.* biopsy). For many years, researchers have investigated computer-aided diagnosis techniques to automatize the workflow and improve accuracy.^3–5^

Nowadays, there is renewed interest in the combination of both quantification and visual assessment to provide a comprehensive quantification of imaging data sets. Instead of reporting only a single quantitative measure or a visual subjective report, image-processing techniques are available to describe many different properties that could be quantified from imaging such as the shape and size of tumours and intensity-based and textural properties with or without additional filtering in a quantitative way. To overcome the wealth of parameters and information, these derived values are combined with statistical modelling techniques to predict a certain clinical end point (*e.g.* survival, local relapse); this field of research is now commonly called radiomics.^6^

**Figure 1.**
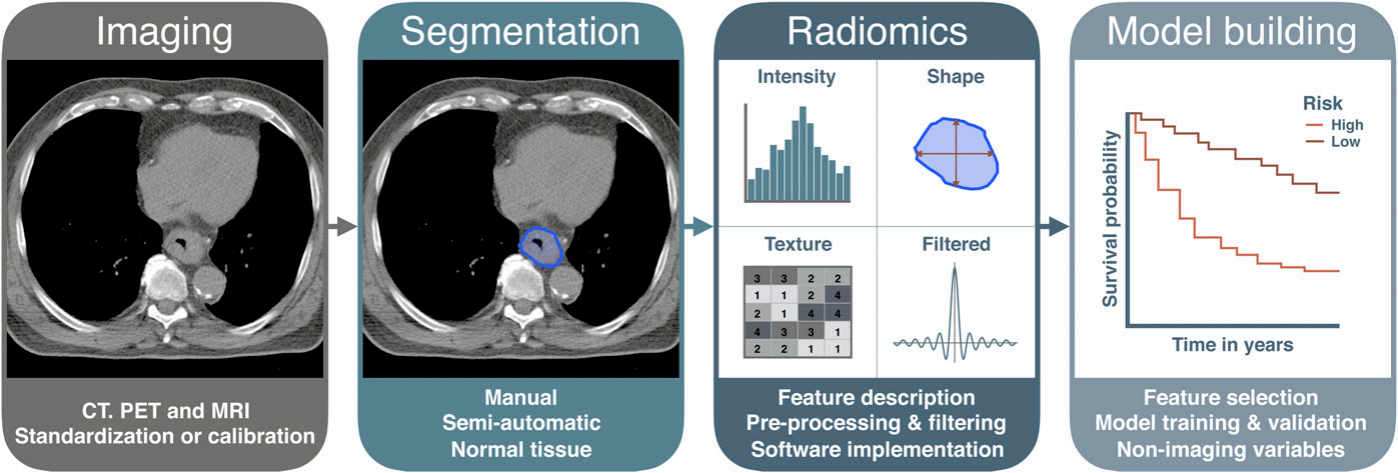
An overview of the radiomics workflow and corresponding topics addressed in this review. PET, positron emission tomography.

The radiomics workflow starts with image acquisition. After image reconstruction, a region of interest is selected that defines the volume for feature extraction. Calculation of these features is performed by image-processing software sometimes including different pre- and post-processing steps. Next, a statistical model is built that allows the selection of features that are able to predict the outcome parameter (*e.g.* survival). Finally, a validation of the model needs to be performed, preferably external validation. An example of the workflow is shown in Figure 1. With this review, we aim to give an overview of the various technical routines and protocols that are involved in quantitative imaging studies such as radiomics. The influence and technical aspects of the above steps will be described in detail in the next sections.

## IMAGE ACQUISITION

Medical images acquired for standard clinical diagnostics, (radiotherapy) treatment planning and follow-up purposes are a source of information for radiomics analyses. In the field of oncology, the most widely used modalities include ultrasound, CT, positron emission tomography (PET) and MRI. Many radiomics studies are relying on retrospective data sets, in which individual image acquisition parameters can be different. These different settings can have an influence on the quality and reliability of the extracted radiomic features, as will be discussed below in detail for the commonly used imaging modalities.

### CT

Several groups investigated the repeatability and robustness of radiomic features in CT scans, showing that the features can have a high test–retest stability^7^ with an acceptable dynamic range.^8^ Mackin et al^9^ scanned a phantom with different parameters on CT scanners of four different manufacturers. They found that variability in textural features calculated on CT scans from different scanners can be in the same order of magnitude as the variability observed in CT scans from patients with non-small-cell lung cancer (NSCLC). Zhao et al^10^ used the publically available Reference Image Database to Evaluate Therapy Response data set,^11^ consisting of 31 patients with NSCLC with same-day repeated CT scans, to assess the impact of slice thickness and reconstruction algorithm on the stability of 89 radiomic features. They concluded that repeatability of features derived from scans with the same imaging settings was good; however, only 19% of the features were repeatable when different settings were used. In cone-beam CT scans, Fave et al^12^ found that radiomic features may be reliable as long as the imaging protocol is consistent and relative differences are used. In addition to all imaging parameters that should be taken into account before performing radiomics analyses, the influence of respiratory motion on radiomic features should not be underestimated. It was shown that up to almost 75% of the CT radiomic features can be susceptible to respiration, making breath-hold or four-dimensional CT acquisition necessary for moving lesions.^13^

### Positron emission tomography

PET scans were used to show a good test–retest stability in up to 71% of the radiomic features in a cohort of patients with NSCLC.^14^ In oesophageal cancer, heterogeneity parameters such as entropy, homogeneity, dissimilarity (local characterization) and variability in the size and intensity of homogeneous tumour areas (regional characterization) also had a good reproducibility.^15^ However, various studies found that PET radiomic features can be susceptible to reconstruction parameters. For instance, Galavis et al^16^ showed that in a cohort of 20 patients with different types of solid tumours, 40 (80%) of the 50 features tested presented large variations (range >30%) when the number of iterations, grid size, reconstruction algorithm and/or post-reconstruction filter was changed. This was also confirmed by van Velden et al^17^ and Yan et al,^18^ who tested the impact of the same reconstruction parameters on feature robustness. Respiratory motion causing blurring is another matter of concern. Yip et al^19^ demonstrated that textural features can vary up to 19% when comparing their values derived from three-dimensional (3D)-PET with those from four-dimensional PET scans. Also, the studies by Oliver et al^13^ and Grootjans et al^20^ showed that respiratory motion has a significant effect on the quantification of tumour heterogeneity with PET. In addition to this, it is shown that the specific way of SUV discretization has a crucial effect on the resulting textural features, and obviously their interpretation, in both oesophageal^21^ and lung carcinomas.^22^ This effect turned out to be comparable in both carbon-11 choline and fluorine-18 fludeoxyglucose (FDG)-PET imaging.^23^ Similar to the current European Association of Nuclear Medicine/EANM Research Ltd harmonization protocol for FDG-PET imaging,^24^ a thorough comparison of imaging and reconstruction parameters is warranted before two independent data sets can be compared with each other in a radiomics analysis.

### MRI

Radiomics on MRI has not been investigated as extensively as on CT and PET scans. Its potential value is shown by, for instance, Wibmer et al^25^ and Gnep et al,^26^ both showing that textural features of prostate MRI may differentiate non-cancerous and cancerous prostate tissues and may correlate with biochemical recurrence and Gleason score. However, to our knowledge, to date, no studies investigating the repeatability and stability of radiomic features in MRI have been published. Given the fact that geometrical distortions are quite common in MRI,^27^ further research investigating its effect on radiomic feature extraction is needed.

### Ultrasound

Quantitative features retrieved from ultrasound images have been mainly shown to be useful to discriminate among normal, malignant and benign tissues.^28^ Andrėkutė et al^29^ evaluated whether acoustical, textural and shape features were able to differentiate malignant melanoma from benign melanocytic tumours. They identified a combination of seven relevant features, yielding an accuracy of 82.4%. Similar accuracies were observed when using quantitative (textural) features to identify malignant thyroid nodules^30,31^ or breast tumours.^32,33^ Although a study by Nadeau et al^34^ revealed a high interobserver variability in quantitative ultrasound features of the Achilles tendons, to date no integrated analysis testing the repeatability and stability of ultrasound radiomic features for applications in oncology has been published.

### Standardization or calibration

Currently, many radiomics studies in different cancer types have used data acquired in a single institute, sometimes combined with an internal validation step.^26,35–40^ These studies showed the great potential for radiomics. However, the lack of standardization/harmonization or at least a correlation between radiomic features acquired in different settings (*e.g.* scanner type, hospital, radiomics software) makes it difficult to directly compare different studies and extracted feature values. This makes blindly interchanging data sets for validation without taking the different acquisition settings into account difficult. Specific radiomic correction and calibration algorithms could potentially solve this issue and are currently being investigated.

For future prospective studies, it could therefore be beneficial to adopt acquisition and reconstruction standards, as proposed by, for instance, the Quantitative Imaging Biomarker Alliance, Quantitative Imaging Network, American Association of Physicists in Medicine and European Association of Nuclear Medicine.^24,41,42^ Nonetheless, standardization can be challenging with the introduction of new, state-of-the-art imaging equipment in different institutes and cannot be applied to the enormous amount of retrospective data available.

## TARGET VOLUME DEFINITION

### Manual segmentation

Accurate segmentation is an important step of the radiomics workflow, as radiomic features are derived from segmented volumes of interest. Manual delineation of the gross tumour volume is a standard clinical routine in the treatment-planning process for patients receiving radiotherapy, but for other interventions, this is not frequently performed. Manual delineation is a straightforward solution, but can also be very time consuming and is susceptible to interobserver variability. For instance, interobserver delineation in cervical cancer can lead to significant differences and are reported to differ up to 4 cm.^43^ These differences can influence radiomic features extracted from the delineated volumes. Van Velden et al^17^ investigated the influence of reconstruction and delineation on the repeatability of radiomic features in patients with NSCLC and concluded that 24% of the features were susceptible to the delineation method. Also, Leijenaar et al^14^ found in a cohort of 23 patients with NSCLC that radiomic features are susceptible to differences in delineation; however, they observed that 91% of the PET features still had a high interobserver stability.

### (Semi-)automatic segmentation

Automatic or semi-automatic segmentation methods are currently investigated extensively to minimize manual input and increase consistency in delineating the regions of interest. In a cohort of patients with hepatocellular cancer, Echegaray et al^44^ compared manual delineations with core samples that were obtained by automatically tracing the maximal circle inscribed in the outlines. They showed that the same set of stable features can be retrieved from the core sample, providing as much information as a detailed segmentation. Balagurunathan et al^45^ showed that most of the radiomic features have a high reproducibility using an automated, seed-based image analysis program with segmentation performed by a single reader. Parmar et al^46^ compared manual slice-by-slice delineations of five physicians with semi-automatic segmentation using the 3D Slicer platform. They showed that radiomic features extracted from 3D Slicer volumes had a significantly higher reproducibility and were more robust than those extracted from manual segmentation. Other groups also reported that semi-automatic segmentation methods have a good correlation with pathology.^47,48^

Depending on the application, more advanced methods such as the fuzzy c-means,^49^ fuzzy hidden Markov chains^50^ or fuzzy locally adaptive Bayesian (FLAB)^51^ segmentation algorithms might be preferred over the discussed methods. Especially for smaller lesions, the FLAB algorithm showed a superior performance when compared with fuzzy c-means or fuzzy hidden Markov chains.^51,52^ Two other studies demonstrated that the FLAB algorithm had a good correlation with pathology^53^ and that measures derived from FLAB-segmented volumes had a significantly higher predictive value in oesophageal cancer than the same measures derived from volumes segmented with a fixed threshold segmentation.^54^

**Figure 2.**
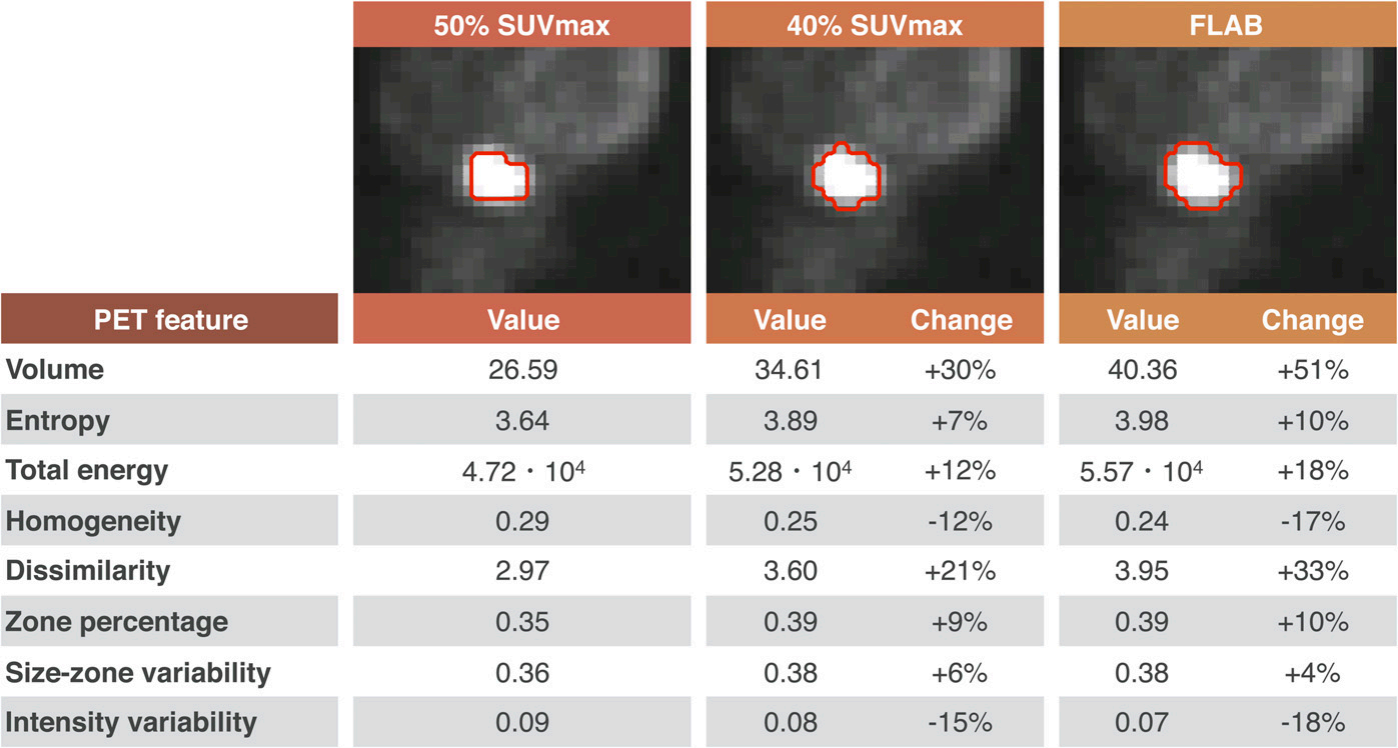
Positron emission tomography (PET) radiomic features^14^ and their dependency on the delineation method in oesophageal cancer: the 50% maximum standardized uptake value (SUV_max_) delineation is used as reference. Fuzzy locally adaptive Bayesian (FLAB) delineation was implemented as described previously.^51^

Although semi-automatic segmentation methods will outperform manual segmentations in terms of repeatability, radiomic features will still be depending on the segmentation method used, as shown in Figure 2. Using fixed thresholds or the FLAB algorithm results in volumes that can differ up to 51%. This obviously influences volume-related features (*e.g.* energy), but also textural features such as grey-level co-occurrence matrix dissimilarity.

### Normal tissue segmentation

The majority of the radiomics studies published to date mainly focused on analyses on malignant lesions for use in prognostic or predictive models. However, radiomics analysis of imaging data might also have other applications. For example, it was shown that a texture-based measure of pulmonary CT scans had a superior performance in discriminating patients with and without Chronic Obstructive Pulmonary Disease when compared with the commonly used densitometric measures.^55^ Using screening CTs of the National Lung Screening Trial,^56^ Hawkins et al^57^ developed a radiomics model consisting of 23 stable features to predict nodules that will become cancerous at 1 and 2 years with accuracies of 80% [area under the curve (AUC 0.83)] and 79% (AUC 0.75), respectively. Also, in patients with early-stage NSCLC who received stereotactic body radiation therapy, a combination of high-risk features on post-stereotactic body radiation therapy CT scans was identified to accurately distinguish between local recurrences and fibrosis.^58^ These features were also validated in an independent patient cohort.^59^ Similarly, Cunliffe et al^60^ analyzed the relationship between radiation dose and development of radiation pneumonitis, with changes in CT radiomic features before and after chemoradiation of patients with oesophageal cancer. They observed a significant change in radiomic features with increasing dose for all features, while for a selection of features this change was significantly related to radiation pneumonitis development. Although not all correlations discussed above are confirmed in independent data sets yet, the first results demonstrate the potential ability of quantitative imaging features to assess the risk for the development of cancer in healthy subjects, local recurrences after initial treatment or radiation-induced side effects in normal tissues.

## FEATURE EXTRACTION

### Radiomic feature description

Radiomic features are derived from the information contained in the voxels of the segmented structure. The features can be grouped into different categories and some typical examples are discussed below. For the full mathematical description, we refer to the respective publications.

First-order statistics are derived from the histogram of voxel intensities (*i.e.* Hounsfield units for CT imaging or SUV values for PET imaging). These histogram characteristics reflect the mean value, dispersion (standard deviation, mean absolute deviation), central moments (skewness and kurtosis describing asymmetry and sharpness, respectively) and randomness (entropy, uniformity).^61^ The area under the cumulative SUV histogram has been used as a heterogeneity measure in FDG-PET scans.^62^

Texture or greyscale variation features, widely used in pattern recognition, refer to higher order statistical measures and summarize the local spatial arrangement of intensities. The textural features are based on different parent matrices capturing this spatial intensity distribution. The grey-level co-occurrence matrix,^63^ counting voxel pairs with certain grey values at a predefined direction and distance from each other, generates the features homogeneity, contrast and sum variance. The neighbourhood grey-tone difference matrix^64^ is based on the differences between each voxel and the neighbouring voxels, resulting in features said to resemble the human perception of the image: coarseness, complexity and texture strength. Similarly, the neighbouring grey-level dependence matrix^65^ considers all neighbouring voxels eliminating angular dependency. Small number emphasis (image fineness) and large number emphasis (image smoothness) are computed from this matrix. Grey-level run length^66^ features such as short and long run emphases focus on collinear voxels with the same grey level value. Finally, grey-level size zone-based features^67^ target groups of connected pixels with the same grey value.

Wavelet decomposition of the original image has been employed to extract intensity and textural features from different frequency bands^61^ and to obtain fused texture characteristics from two imaging modalities.^68^ All intensity-based and textural features were mostly calculated in a volumetric way (*i.e.* all voxels within the region of interest were taken into account), with some studies considering only one image slice in the calculation (mostly the slice with the largest cross-sectional area).^69^ For the volumetric features, slice thickness clearly impacts on feature extraction results. The nature of matrix-based textural features makes them highly sensitive to the present voxel size anisotropy. Some studies therefore performed a resampling to an isotropic voxel size as a pre-processing step before feature extraction.^68^ Others compared the volumetric and in-slice approaches and concluded that the largest cross-sectional area analysis was an acceptable substitute for volumetric analysis for many features. However, in order to avoid potential erroneous conclusions by overlooking parts of the tumour this way, the use of volumetric features incorporating information of the whole tumour was recommended.^70,71^

Shape-based features describe the 3D geometrical composition of the segmented structure. Size (volume and maximal diameter) and shape measures (sphericity, compactness and surface-to-volume ratio) have been described in radiomics research.^37,61^ Finally, features relating to tumour location (*e.g.* lung lobe, distance to the organ at risk) have been used.^72^

### Image pre-processing and filtering

Most radiomics studies have used the reconstructed CT data available in the digital imaging and communications in medicine files, without any pre-processing or normalization.^61^ Possible image pre-processing includes image smoothing by average or Gaussian filters reducing image noise and image enhancement techniques such as histogram equalization, deblurring and resampling.^68,73^ Image filtering has been performed, *e.g.* using a Gaussian filter followed by a Laplacian on the image, resulting in so-called Laplacian of Gaussian features.^37,74^

Computation of textural features requires discretization of the intensity values. Aerts et al^61^ used equally spaced 25-HU bins for CT data. In FDG-PET data, Tixier et al^15^ described the sensitivity of several textural features to varying discretization values, while Leijenaar et al^22^ have shown that a fixed bin resolution enhances the interpatient and intrapatient reproducibility of feature values.

### Software implementation

Several open-source software packages are available to conduct radiomics analyses. The “imaging biomarker explorer” was specifically designed to facilitate collaborations in radiomics research, offering standardized settings for image pre-processing and feature extraction.^73^ This addresses the important need for benchmarking in the rapidly growing radiomics field.^75^ The “Chang Gung Image Texture Analysis”^76^ offers FDG-PET image analysis, while the MaZda package was originally designed for MR images.^77^ The commercial packages RADIOMICS^™^ (OncoRadiomics, Maastricht, Netherlands) and TexRAD^™^ (Feedback plc, Cambridge, UK) have been developed. An overview of the main functionalities of these packages is presented in Table 1. Several in-house implementations exist as well, *e.g.* coded in MATLAB^®^ (The Mathworks^®^, Natick, MA) within the Computational Environment for Radiotherapy Research environment.^37,78^

**Table 1.** Overview of currently available software packages for radiomics analysis (August 2016)

Software package	Imaging modality and format	ROI definition	Features and image pre-processing	Model building	Website
IBEX (free open source)	CT, PET, MR DICOM, Pinnacle native format	DICOM-RT Editing and free drawing	109: intensity, texture, shape Smoothing, resampling, enhancement	Validation of existing models	http://bit.ly/IBEX_MDAnderson
CGITA (free open source)	Designed for PET; CT, MR tested-DICOM	DICOM-RT, PMOD Region growing and FCM	72: intensity, texture No pre-processing	None	http://code.google.com/p/cgita
MaZda (free open source)	Designed for MR DICOM	Thresholding, deformable surface	279: intensity, texture, shape, wavelet Resampling, discretization, normalization	ANN, clustering Fisher, MI, PCA	http://www.eletel.p.lodz.pl/programy/mazda/
RADIOMICS^™^ (OncoRadiomics, Maastricht, Netherlands) (commercial)	CT, PET, MR DICOM	DICOM-RT Plug-in for several TPS	543: intensity, texture, shape, wavelet Laplacian of Gaussian Resampling, discretization	None	http://www.oncoradiomics.com
TexRAD^™^ (Feedback plc, Cambridge, UK) (commercial)	CT, PET, MR DICOM	DICOM-RT Editing, thresholding	−30: texture and filtering (Laplacian of Gaussian)	Data-mining tool	http://www.texrad.com

ANN, Artificial Neural Network; CGITA, Chang Gung Image Texture Analysis; DICOM, digital imaging and communications in medicine; DICOM-RT, Digital Imaging and Communications in Medicine-Radiation Therapy; FCM, fuzzy c-means; IBEX, imaging biomarker explorer; MI, Mutual Information; PCA, Principal Component Analysis; PET, positron emission tomography; PMOD, PMOD Technologies LLC; ROI, region of interest; TPS, Treatment Planning System.

Their characteristics and functionalities for the four main steps of the radiomics workflow are summarized.

## VALIDATION AND MODEL BUILDING

### Feature selection methods

Typically, the number of calculated image features is much larger than the sample size of patients studied. Dimensionality reduction is therefore crucial to reduce the risk of overfitting by focusing the attention of subsequent classification efforts on a subset of relevant features. As most of the features, being based on the same matrix or quantities, will exhibit some correlation with each other, intelligent feature selection strategies are required.

Filter-based selection techniques of the univariate type (*e.g.* Relief,^79,80^ Fisher score and Wilcoxon) select informative features, while multivariate filter techniques (*e.g.* minimum redundancy maximum relevance^37^ and mutual information) take redundancy into account as well. In a study comparing 14 filter-based feature selection techniques in combination with 18 machine-learning (ML) classification procedures in radiomics cohorts, Wilcoxon test-based feature selection showed the highest stability against data perturbation in combination with most classifiers.^81^ Wrapper selection techniques are tailored to a specific classification algorithm with the algorithm being part of the subset evaluation.^80,82^

Principal component analysis performs a transformation for dimensionality reduction and can highlight outliers.^72,83^ Aerts et al^61^ have used the single most informative of the four studied feature categories to reduce redundancy. The false discovery rate^37,84^ has been used to increase testing power compared with the conservative Bonferroni correction. The dynamic range of feature values can also be an important selection criterion.^72^

### Model training and validation

Ultimately, the goal of most radiomics analyses is to obtain a prognostic or predictive model with a high accuracy and efficiency. Ideally, enough data are available to train and validate a classification model while holding out part of the data for the assessment of accuracy (external validation).

Unsupervised ML analyses using heat maps or clustering summarize feature data without involving an outcome variable.^85^ Supervised ML classifiers such as generalized linear models, random forests,^86^ support vector machines^87^ and neural networks separate the data with respect to an outcome variable. Among the 18 supervised ML classification procedures studied by Parmar et al,^81^ random forests had the highest performance in combination with most feature selection techniques. Moreover, the choice of classification method was found to be the dominant source of performance variation. The number of features selected as input to the classification models had a negligible impact, explaining <2% of the total variance.

Regularization methods control the complexity of a prediction model to prevent overfitting. The Lasso technique, using L1 regularization, was applied in combination with logistic regression and Cox regression (optimizing time to outcome).^88,89^ It presents a weak dependence on irrelevant features, in contrast with L2 regularized logistic regression (and also with support vector machines and neural networks), which shows in worst case a linear relation between the number of irrelevant features and the required sample size.^90^

Cross-validation has frequently been used as internal validation with the AUC of the receiver-operating characteristic curve (or its generalization, concordance index) evaluating the performance accuracy.^7,37,91^

Finally, the understandability of classification models varies significantly and should be considered,^72^ together with qualitative reporting.^92^

### Integration of non-imaging variables

Conventional image-derived features such as tumour volume or diameter and maximum SUV have been used as reference models in radiomics studies.^61^ Incorporating the most informative radiomic features into these reference models provides an indication of the gain in model performance. Patient and clinical characteristics might be added to the input variable list, as they potentially influence not only the outcome variable, but also the extracted radiomic features themselves.^72,89^

## FUTURE OUTLOOK

Although the concept of using quantitative parameters describing imaging data is not new, the combined application in the radiomics workflow is currently under investigation by many research groups and has shown to be of added value. This renewed interest is mainly driven by the increased digitalization in the hospital, introduction of electronic medical records and easier access to large amounts of information through the hospital picture archiving and communication systems combined with the increased computational power. In this era of Big Data, in medical research fields also, a new field of clinical data science is emerging that allows the integration of multiple data sources to personalize treatment.^93^ Radiomics is expected to be a critical component for the integration of image-derived information in this framework. Currently, imaging research is typically performed retrospectively on data stored in the picture archiving and communication systems. As described in this review, an investigation on the quality of the input imaging data is necessary to assess the potential influence on outcome prediction accuracy.

There is, however, a concern related to the use of image-derived features as such in the individualization of patient care. Treatment interventions need to be optimized and frequently (not always) this is performed on a biological or methodological basis where two treatments are compared with each other. Radiomics quantifies the phenotype of the underlying biology; however, this link is not straightforward.^94,95^ More research is needed into the correlation between the underlying biological processes and the perceived image data sets. If this link is established, treatment can be tailored to the individual patient based on the imaged properties of tumours. This integration with biology might then also allow for hypothesis-driven research that is still necessary to improve the methodological understanding of the biological processes. Furthermore, a reliable predictive or prognostic power is necessary if implemented in clinical routine for individual decision-making.

## FUNDING

This work was partly funded by the European Union Seventh Framework Programme for research, technological development and demonstration under grant agreement number 601826.
